# A Hybrid 2D Gaussian Filter and Deep Learning Approach with Visualization of Class Activation for Automatic Lung and Colon Cancer Diagnosis

**DOI:** 10.1177/15330338241301297

**Published:** 2024-12-04

**Authors:** Omer Turk, Emrullah Acar, Emrah Irmak, Musa Yilmaz, Enes Bakis

**Affiliations:** 1 Faculty of Engineering and Architecture, Department of Computer Engineering, Mardin Artuklu University, Mardin, Turkey; 2 Faculty of Engineering and Architecture, Department of Electrical and Electronics Engineering, 187432 Batman University, Batman, Turkey; 3 Faculty of Engineering, Department of Electrical and Electronics Engineering, 450199 Alanya Alaaddin Keykubat University, Antalya, Turkey; 4 Bourns College of Engineering, Center for Environmental Research and Technology, University of California at Riverside, Riverside, CA, USA; 5 Faculty of Engineering, Department of Electrical and Electronics Engineering, 187480 Piri Reis University, Istanbul, Turkey

**Keywords:** lung and colon cancer, Gaussian (Blur) filter, ResNet50, deep learning

## Abstract

Cancer is a significant public health issue due to its high prevalence and lethality, particularly lung and colon cancers, which account for over a quarter of all cancer cases. This study aims to enhance the detection rate of lung and colon cancer by designing an automated diagnosis system. The system focuses on early detection through image pre-processing with a 2D Gaussian filter, while maintaining simplicity to minimize computational requirements and runtime. The study employs three Convolutional Neural Network (CNN) models-MobileNet, VGG16, and ResNet50-to diagnose five types of cancer: Colon Adenocarcinoma, Benign Colonic Tissue, Lung Adenocarcinoma, Benign Lung Tissue, and Lung Squamous Cell Carcinoma. A large dataset comprising 25 000 histopathological images is utilized. Additionally, the research addresses the need for safety levels in the model by using Class Activation Mapping (CAM) for explanatory purposes. Experimental results indicate that the proposed system achieves a high diagnostic accuracy of 99.38% for lung and colon cancers. This high performance underscores the effectiveness of the automated system in detecting these types of cancer. The findings from this study support the potential for early diagnosis of lung and colon cancers, which can facilitate timely therapeutic interventions and improve patient outcomes.

## Introduction

Cancer is one of the most researched diseases that endanger human health and has the highest mortality rate among all diseases.^1^ Tumors are generally of two types.^2^ Among these, benign tumors are not cancerous, they can be removed with an easy surgical procedure, and they can rarely be dangerous because they do not recur frequently. Malignant tumors, on the other hand, are dangerous because they grow irregularly and uncontrollably and cause cancer.^3^ Cancer is a significant reason of death worldwide with approximately 10 million deaths in 2020. The most common cancer types in terms of causing the deaths in 2020 were lung [1.80 million deaths], colon and rectum [935 000 deaths], liver [830 000 deaths], stomach [769 000 deaths] and breast [685 000 deaths].^4^ When these statistical data are considered, the first two cancer types that cause the most deaths in 2020 are colon and lung cancers. It has been proven by clinical studies that cancer can be treated when detected early. Therefore, early diagnosis of lung and colon cancers, as in other cancers, is extremely important for clinicians and researchers interested in this field. The reason why both cancers are diagnosed together is that the probability of metastasis between the two organs is higher, rather than the simultaneous emergence of both cancers.

In recent years, the scientists have developed many approaches for the early detection of cancer. One of these approaches is the use of medical imaging methods, which play a very effective and important role in the cancer detection.^5^ However, early detection of cancer becomes a correspondingly difficult task, as the manual interpretation of medical imaging data takes some time. In addition, misinterpretation of medical images by experts prolongs the period of early diagnosis of cancer disease and accordingly decreases the accuracy rate.^6^ In order to overcome this problem, many machine learning-based algorithms have been employed in the literature and different cancer types have been detected early with satisfactory accuracy.

Considering the studies in the literature, two main approaches based on machine learning,^7^ which constitute the sub-branch of artificial intelligence, have been utilized for the diagnosis of lung and colon cancers. The first of these covers Conventional Machine Learning (CML) algorithms. These algorithms are usually employed together with predefined features, which are obtained by feature extraction methods.^3^ Some of the studies in the literature carried out for the diagnosis of colon and lung cancer by utilizing CML-based algorithms can be summarized as follows: Xu et al have proposed a model to classify colon cancer images via classical feature extraction methods and multi-class SVM classifier. Their results showed that the average precision of 73.7% has been observed for classification of colon cancer images.^8^ Shi et al have recommended a novel approach in order to classify lung needle biopsy images by employing multimodal sparse representation-based classification technique. Finally, they have achieved important advancement with average accuracy of 88.1% for classifying different lung cancerous types.^9^ Kuruvilla and Gunavathi have proposed an approach for early detection of lung cancer by utilizing feed forward and feed forward back propagation neural networks via statistical features. The accuracy result of their proposed approach has been found as 93.3%.^10^ Hussain et al have developed a tool in order to detect lung cancer automatically by utilizing multimodal features and various ML techniques (NB, DT and SVM). At the result, they have reached a high accuracy rate for lung cancer detection.^11^ Selvanambi et al have suggested a new work in order to diagnose and predict the lung cancer thanks to the Recurrent Neural Network (RNN) with Levenberg–Marquardt and the glowworm swarm optimization technique. Their experimental result has achieved classification accuracy of 98%.^12^ Naeem et al have successfully detected colon cancer using genomic signal processing approach considering the cancer to be a genetic disease. In the proposed study, KNN and SVM have been used to classify the statistical features which had been obtained from DNA sequences.^13^

Another efficient approach for diagnosis of lung and colon cancers covers DL based algorithms. In this approach, DL algorithms do not need any predefined features in contrast to CML algorithms. This situation makes the performance of DL approach more superior than CML approach since CML algorithms are inflexible, unstable, and time-consuming when they are manually designed in lung and colon cancer detection. Thus, many CML algorithms have been substituted by DL algorithms in recent years.^3^ Some of the recent works in the literature on the diagnosis of lung and colon cancer using DL based algorithms can be summarized as follows: Masud et al have proposed a Convolutional Neural Network (CNN) based classification method to classify different types of lung and colon cancer tissues. They have obtained an overall classification accuracy of 96.33%.^14^ While the method used in this study has given good results for three disease groups, it achieved low classification success for the other two. In addition, Discrete Fourier transform (DFT) and Discrete Wavelet transform (DWT) used for feature extraction are algorithms that require high computational requirements and long working time. Togacar has recommended an approach of DarkNet-19 model with Manta Ray Foreign and Equilibrium optimizations for the classification of lung and colon cancer types. The results have showed a high classification accuracy of around 99%.^15^ Similar to the previous study, an optimization algorithm that increases the hardware requirements has been used in this study. Ali et al have suggested a new multi-input capsule network by employing two convolutional layer blocks to classify lung and colon tumors into five categories. As a result, an overall accuracy of over 99% has been obtained with the proposed approach.^16^ Although the success rate has been increased by using two convolutional layer blocks and giving two inputs to these blocks, the complexity of the whole system stands out as a striking detail. Wang et al have used a novel two-step path for the diagnosis of colorectal cancer using CNN and transfer learning approach. They have achieved an area under the curve (AUC) value of 0.988 using a fairly large dataset.^17^ Garg et al have used 8 well-known pre-trained CNN models to predict lung and colon cancer using histopathological images.^18^ Mehmood et al have successfully detected the malignancy in lung and colon histopathology images making use of a highly accurate and computationally efficient CNN model.^19^ Although the system used is hardware efficient, transfer learning has limited flexibility, overfitting and limited generalization disadvantages. Mridha et al have used four CNN models to detect lung and colon cancers.^20^ Now that each CNN model solves a separate classification problem, indeed a binary classification has been performed rather than a multi-classification. Teramoto et al have diagnosed three lung cancer types using a novel CNN model and obtained an overall accuracy of 71%.^21^ Experiments on colon cancer diagnosis were not conducted. Kumar et al have made use of DenseNet-121 model and handcrafted features to classify lung and colon cancer disease. The authors have obtained an overall accuracy of 98.6%.^22^ The researchers who are interested in the field of lung and colon cancer diagnosis using DL techniques with histopathological images can also refer to^23^ and^3^ which are rich and up-to-date review papers.

Lung and colon cancers are among the most prevalent and lethal forms of cancer globally, underscoring the vital importance of prompt and precise diagnosis for enhancing survival outcomes. The current diagnostic methods, including traditional imaging techniques and manual assessment, are often time-consuming, susceptible to human error, and frequently necessitate the involvement of highly trained professionals. Notwithstanding the advances made in automated diagnostic systems, there remains a considerable gap in the development of efficacious and dependable models that are capable of integrating both feature extraction and interpretability, particularly in the context of intricate medical datasets. This study aims to address this gap by proposing a hybrid approach combining a two-dimensional Gaussian filter with deep learning, along with class activation visualization to enhance the model's interpretability. The objective of this approach is to provide an automated, accurate, and explainable solution for diagnosing lung and colon cancer, which is of great significance in improving clinical outcomes and facilitating early intervention. In addition, the motivation of this study is to diagnose lung and colon cancers from the histopathological images using DL models that have been trained through a fairly large dataset. Three deep learning models (MobileNet, VGG16 and ResNet50), which have been used and yielded very successful results many times in the literature in the diagnosis of medical diseases from medical images so far, have been used for the purpose of lung and colon cancer detection. In order for the DL models to yield more successful results, all histopathological images have been passed through a 2D Gaussian (Blur) filter and the images have been made more suitable for further processing. The regions of the image learned by the CNN network during the learning process have been visualized with the Class Activation Map (CAM) technique. This study is believed to be an important aid to doctors and radiologists in diagnosing lung and colon cancer.

When the experimental results obtained are taken into account together with similar studies and results in the literature, the main contribution of this study can be summarized as follows:

✓ The proposed system in this study makes it possible to speed up the detection of lung and colon cancer by enabling doctors or radiologists to examine a large number of patients in a shorter time and a lower cost.
✓ The proposed CAD system is able to diagnose lung and colon cancer with a high accurate rate such as 99.38%.
✓ Using 2D Gaussian Filter as a pre-processing stage prior to training highly increases the overall accuracy rate.
✓ Even in classifying images of the same organ, the ResNet50 model achieves high success.
✓ The proposed method can be used for mobile phone-based medical disease diagnosis as it uses the MobileNet model and is compatible with mobile vision.

The rest of this paper can be organized as follows: Section II introduces the materials, methods and dataset that has been used. Section III presents and discusses the results of the experiments conducted and finally Section IV concludes the paper.

## Materials & Methods

### Dataset Description & Data Augmentation

**Figure 1.**
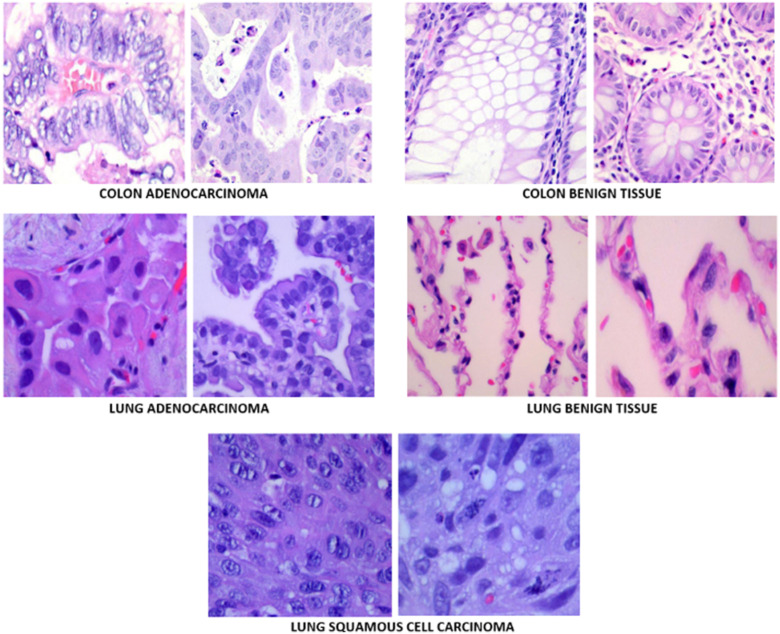
Sample images in the dataset.

One of the most important factors for success in DL studies is the dataset used. In the proposed study, Lung and Colon Cancer Histopathological Images dataset (LC25000)^24^ has been used. This is a publicly and freely available dataset that can be downloaded from a public database.^25^ The dataset contains cancerous and benign lung and colon tissue images. It totally includes 1250 histopathological images and is divided into 5 groups as 250 benign lung images, 250 lung adenocarcinoma, 250 lung squamous cell carcinomas, 250 benign colon tissue 250 colon adenocarcinomas. The LC25000 dataset images were collected at James A. Haley Veterans’ Hospital situated in Tampa, Florida. All the images were acquired from pathology glass slides using a Leica Microscope MC190 HD Camera connected to an Olympus microscope and fulfill the requirement for the Health Insurance Portability and Accountability Act (HIPAA). Although the original size of the images was 1024 × 768, they have been re-sized to 768 × 768 to be more compatible inputs to DL models. Images were carefully checked and annotated by an expert pathologist to make sure the quality of images and annotations. The number of images in the dataset is not sufficient for a valid and successful machine learning study. Therefore, to increase the generalization ability of DL models the original dataset has been augmented using vertical and horizontal flips (0.5 probability), and left and right rotations up to 25 degrees. After data augmentation there are 25 000 images in total and 5000 in each group. Figure 1 shows sample images from the dataset and Table 1 shows number of images used in this study before and after data augmentation.

**Table 1.** Data Augmentation.

Image Type	Diagnosis Type	Number of Images before Data Augmentation	Number of Images after Data Augmentation
Colon Images	Colon Adenocarcinoma	250	5000
	Benign Colonic Tissue	250	5000
Lung Images	Lung Adenocarcinoma	250	5000
	Benign Lung Tissue	250	5000
	Lung Squamous Cell Carcinoma	250	5000

### Proposed Method

**Figure 2.**
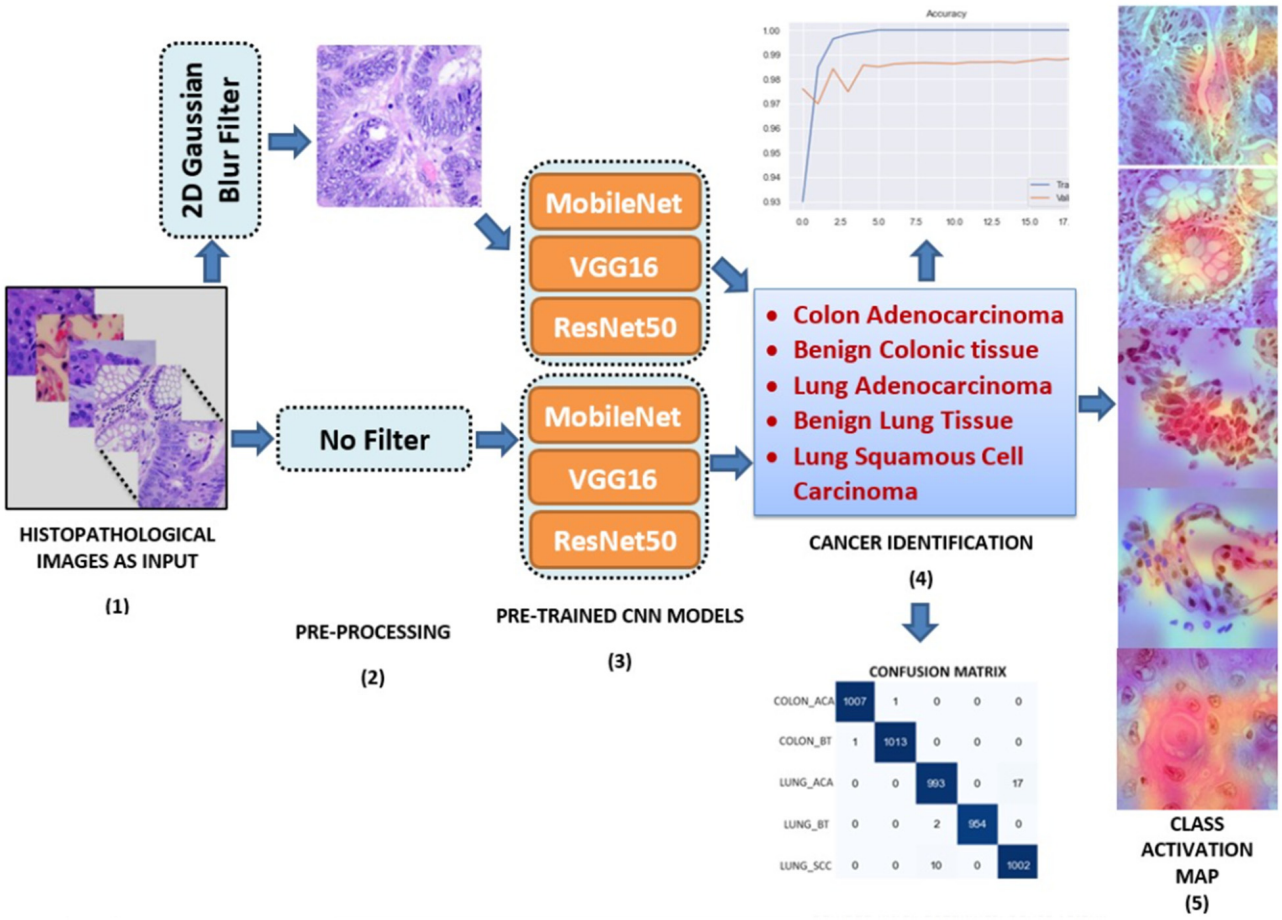
Graphical representation of the proposed algorithm.

Manual evaluation of medical images takes a lot of time, requires specialists and is prone to error, which shows the importance of diagnosing lung and colon cancer by DL based CAD systems. This study proposes a CAD system to diagnose lung and colon cancer from histopathological images using CNN method. A quick review of literature on medical disease diagnosis from medical images shows that the most efficient DL method used for this purpose is CNN method.^26,27^ It is possible to find many studies that successfully diagnose diseases from medical images using CNN models.^28,29^ Since the CNN method has proven itself in this field, the CNN method has been used for the detection of lung and colon cancer in this study. Figure 2 is the general layout of the proposed method. The proposed method uses three popular CNN models which are MobileNet, VGG16 and ResNet50. These models have been first trained on a large dataset such as 25 000 histopathological images. 2D Gaussian (Blur) filter has been used as a pre-processing stage to denoise the input images. As soon as an input image is fed to the proposed algorithm, it is gone under feature extraction and classification processes and a decision is made whether it is Colon Adenocarcinoma, Benign Colonic Tissue, Lung Adenocarcinoma, Benign Lung Tissue and Lung Squamous Cell Carcinoma. The performance of the proposed method has been evaluated using performance evaluation metrics derived from confusion matrix. Finally, in the last stage, the prediction decision made by the CNN has been interpreted by CAM visualization technique.

### 2D Gaussian (Blur) Filter

The Gaussian blur filter^30^ was selected for its capacity to efficiently diminish noise and detail in images while maintaining essential features, rendering it an optimal pre-processing instrument for medical image analysis. In the context of lung and colon cancer diagnosis, medical images frequently contain artefacts or irrelevant details that can impede the performance of deep learning models. The application of a Gaussian blur filter serves to mitigate the impact of minor inconsistencies, thereby enhancing the overall clarity of the image, particularly in regions of interest such as tumour boundaries. Furthermore, the application of a Gaussian blurring function introduces a controlled level of smoothing, which serves to emphasise the larger structures of interest without completely eliminating the edges that are critical for accurate feature extraction. This makes the filter particularly suitable when combined with deep learning models, as it enhances the model's ability to focus on relevant features, thereby potentially improving classification accuracy in complex medical datasets.

**Figure 3.**
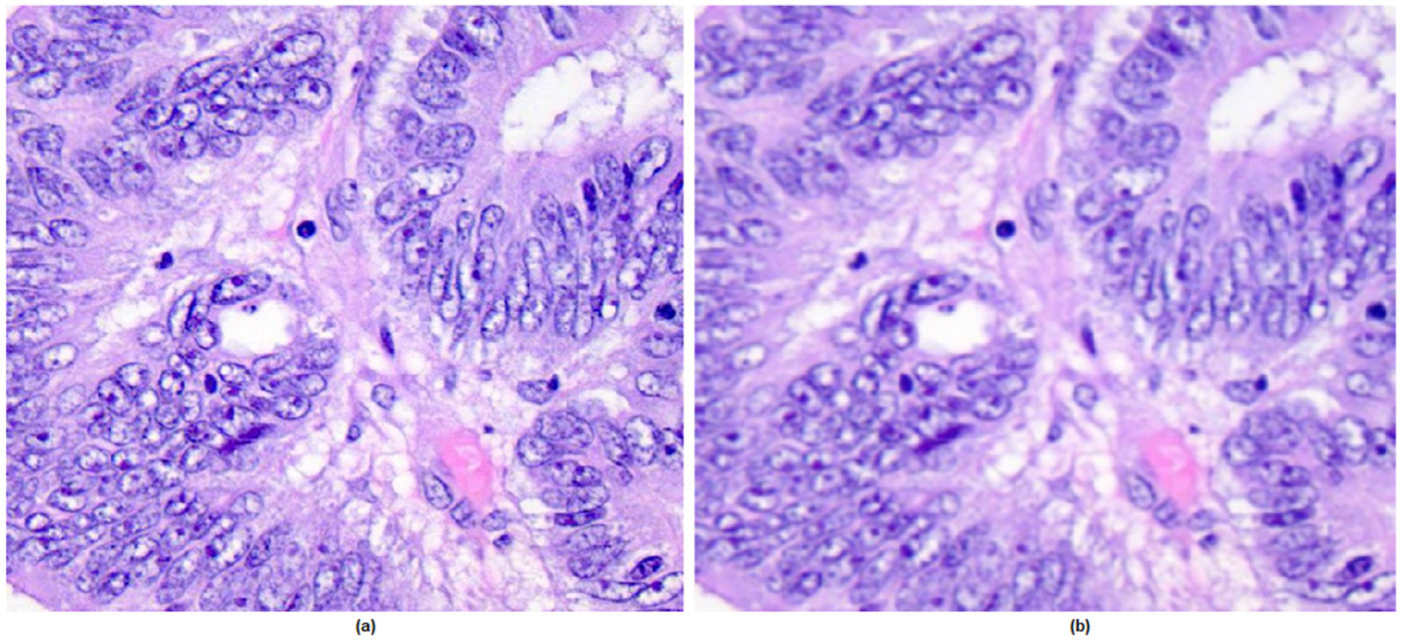
(a) an image of the original example (b) an example image filtered with 2D Gaussian filter.

The two-dimensional Gaussian function that is used in filtering can be computed as follows:

(1) $$G(x,y)=\frac{1}{2\pi \sigma^{2}}e^{-\frac{x^{2}+y^{2}}{2\sigma }}\;$$

where x and y are location indices and σ is standard deviation of the function. This distribution produces a convolution matrix that will be applied to input image. Gaussian filter size used in this study is 11 × 11. High-frequency unwanted noises, such as sharp edges and dots, appear in the images while down-sampling an image to make them suitable for DL models. Therefore, Gaussian Blur Filter is a suitable pre-processing step prior to learning process. The original image and a sample 2D Gaussian filtered image are shown in Figure 3.

### Deep Learning Approaches

Advances in DL methods have made DL based medical disease diagnosis more objective and faster than the diagnosis made manually by a doctor or radiologist. Considering the remote areas where doctor and hospital facilities are very scarce, it can be understood how important DL based medical disease diagnosis methods are. As mentioned in detail in the literature review, the most commonly used DL method for diagnosis of medical disease is CNN models. Despite the large number of different CNN models developed so far, the substantial function and structure of all CNN models basically consist of two main structures that we distinguish as *feature extraction* and *classification*. CNN models basically perform *feature extraction* and *classification* using five types of consecutive convolutional neural network layers. These are *the input layer*, which receives the input images and makes them suitable for deep learning (resizing and denoising), *the convolution layer* and *pooling layer*, which performs feature extraction, and finally, *the fully connected layer* and *classification layer*, which performs the classification process.

#### MobileNet

MobileNet^31^ is a popular light-weight CNN model with low latency that requires less memory and computational effort. Although MobileNet was first developed and used mostly for DL based mobile applications, its fast speed and minimal utilization of computer power have made it popular for many other applications as well. MobileNet suggests that the depth and spatial dimension (height and width) of a filter used in convolutional layers can be separated, therefore the main characteristic of MobileNet architecture is structure of a depthwise convolution followed by a pointwise convolution. MobileNet model architecture has 28 convolutional neural network layers in total, considering depthwise and pointwise convolutions as separate layers. Considering the possibility and potential of making cancer diagnosis possible with mobile devices, the MobileNet CNN model has been used in this study. The structural details of the MobileNet CNN architecture are given in Table 2.

**Table 2.** Structural Details of MobileNet CNN Model.

* 224 × 224 × 3 input layer
* 1x convolutional layer with 32 chanel and 1x convolutional layer with 64 chanel.
* 1x depthwise convolutional and 2x batchnormalization and 2x ReLu layers with 32 chanel and 1x batchnormalization and 1x Relu layers with 64 chanel.
* 1x zeropadding layer and 1x depthwise convolutional layer with 64 chanel and 1x convolutional layer with 128 chanel
* 1x batchnormalization and 1x Relu layers with 64 chanel and 1x batchnormalization and 1x Relu layers with 128 chanel.
* 1x depthwise convolutional layer, 2x batchnormalization layers, 2x ReLu and 1x convolutional layers with 128 chanel.
* 1x zeropadding layer and 1x depthwise convolutional layer with 128 chanel and 1x convolutional layer with 256 chanel.
* 1x batchnormalization and 1x Relu layers with 128 chanel and 1x batchnormalization and 1x Relu layers with 256 chanel.
* 1x depthwise convolutional, 2x batchnormalization layers, 2x ReLu and 1x convolutional layers with 256 chanel.
* 1x zeropadding layer and 1x depthwise convolutional layer with 256 chanel and 1x convolutional layer with 512 chanel.
* 1x batchnormalization and 1x Relu layers with 256 chanel and 1x batchnormalization and 1x Relu layers with 512 chanel.
* 5x depthwise convolutional, 10x batchnormalization layers, 10x ReLu and 5x convolutional layers with 512 chanel.
* 1x zeropadding layer and 1x depthwise convolutional layer with 512 chanel and 1x convolutional layer with 1024 chanel.
* 1x batchnormalization and 1x Relu layers with 512 chanel and 1x batchnormalization and 1x Relu layers with 1024 chanel.
* 1x depthwise convolutional, 1x batchnormalization layers, 1x ReLu and 1x convolutional layers with 1024 chanel.
* flatten layer, dense layer 1 and dense layer 2

#### VGG16

Developed by the Visual Geometry Group (VGG), VGG16^32^ suggests enhancing network performance by increasing the network depth. The VGG16 CNN model consists of roughly 21 convolutional neural network layers, 13 of which are *convolutional*, 5 are *max pooling*, and 3 are *fully connected*. VGG16 is a deep CNN model that consists of blocks formed by cascading *convolutional* and *max pooling* layers. The number of filters in the first block is 64, and this number doubles in each subsequent block, eventually reaching 512. As the number of filters increases in parallel with the model depth, the number of parameters in the subsequent layers increases significantly. A small kernel such as 3 × 3 with stride size of 1 is used in all convolutional layers of VGG16 CNN model. Therefore, VGG16 is able to extract sparse features remarkably, making it an efficient model to diagnose lung and colon cancers. The structural details of the VGG16 CNN architecture are shown in Table 3.

**Table 3.** Structural Details of VGG16 CNN Model.

* 224 × 224 × 3 input layer
* 2x convolutional layers and 1x maxpooling layer with 64 chanel.
* 2x convolutional layers and 1x maxpooling layer with 128 chanel.
* 3x convolutional layers and 1x maxpooling layer with 256 chanel.
* 3x convolutional layers and 1x maxpooling layer with 512 chanel.
* 3x convolutional layers and 1x maxpooling layer with 512 chanel.
* flatten layer, dense layer 1 and dense layer 2

#### Resnet50

Resnet50^33^ is a 50-layer network trained on the ImageNet dataset and uses convolutional layers with size 1 × 1, 3 × 3, etc instead of 2 convolutional layers with size 3 × 3. An important point here is that more AI layers do not always generate more performance because very deep networks are prone to performance degradation. ResNet model aims to solve the degradation problem of CNN networks using Residual blocks. The degradation problem arises when deep networks begin to converge. As the network depth increases, its efficiency (accuracy) saturates (as expected) but then tends to decline rapidly. ResNet adds shortcuts between layers by connecting the shallow layers and deep layers directly to solve this problem. This simple idea avoids degradation as the network deepens. The structural details of the VGG16 CNN architecture are demonsrated in Table 4.

**Table 4.** Structural Details of MobileNet CNN Model.

* 224 × 224 × 3 input layer
* 1x zeropadding layer with 3 chanel
* 3x convolutional layers, 3x batchnormalization layers and 3x Relu layers with 64 chanel
* 2x convolutional layers, 2x batchnormalization layers, 1x Relu layer and 1x add layer with 256 chanel
* 2x convolutional layers, 2x batchnormalization layers and 2x Relu layers with 64 chanel
* 1x convolutional layer, 1x batchnormalization layer, 1x Relu layer and 1x add layer with 256 chanel
* 2x convolutional layers, 2x batchnormalization layers and 2x Relu layers with 64 chanel
* 1x convolutional layer, 1x batchnormalization layer, 1x Relu layer and 1x add layer with 256 chanel
* 2x convolutional layers, 2x batchnormalization layers and 2x Relu layers with 128 chanel
* 2x convolutional layers, 2x batchnormalization layers, 1x Relu layer and 1x add layer with 512 chanel
* 2x convolutional layers, 2x batchnormalization layers and 2x Relu layers with 128 chanel
* 1x convolutional layer, 1x batchnormalization layer, 1x Relu layer and 1x add layer with 512 chanel
* 2x convolutional layers, 2x batchnormalization layers and 2x Relu layers with 128 chanel
* 1x convolutional layer, 1x batchnormalization layer, 1x Relu layer and 1x add layer with 512 chanel
* 2x convolutional layers, 2x batchnormalization layers and 2x Relu layers with 128 chanel
* 1x convolutional layer, 1x batchnormalization layer, 1x Relu layer and 1x add layer with 512 chanel
* 2x convolutional layers, 2x batchnormalization layers and 2x Relu layers with 256 chanel
* 2x convolutional layers, 2x batchnormalization layers, 1x Relu layer and 1x add layer with 1024 chanel
* 2x convolutional layers, 2x batchnormalization layers and 2x Relu layers with 256 chanel
* 1x convolutional layer, 1x batchnormalization layer, 1x Relu layer and 1x add layer with 1024 chanel
* 2x convolutional layers, 2x batchnormalization layers and 2x Relu layers with 256 chanel
* 1x convolutional layer, 1x batchnormalization layer, 1x Relu layer and 1x add layer with 1024 chanel
* 2x convolutional layers, 2x batchnormalization layers and 2x Relu layers with 256 chanel
* 1x convolutional layer, 1x batchnormalization layer, 1x Relu layer and 1x add layer with 1024 chanel
* 2x convolutional layers, 2x batchnormalization layers and 2x Relu layers with 256 chanel
* 1x convolutional layer, 1x batchnormalization layer, 1x Relu layer and 1x add layer with 1024 chanel
* 2x convolutional layers, 2x batchnormalization layers and 2x Relu layers with 256 chanel
* 1x convolutional layer, 1x batchnormalization layer, 1x Relu layer and 1x add layer with 1024 chanel
* 2x convolutional layers, 2x batchnormalization layers and 2x Relu layers with 512 chanel
* 2x convolutional layers, 2x batchnormalization layers, 1x Relu layer and 1x add layer with 2048 chanel
* 2x convolutional layers, 2x batchnormalization layers and 2x Relu layers with 512 chanel
* 1x convolutional layer, 1x batchnormalization layer, 1x Relu layer and 1x add layer with 2048 chanel
* 2x convolutional layers, 2x batchnormalization layers and 2x Relu layers with 512 chanel
* 1x convolutional layer, 1x batchnormalization layer, 1x Relu layer and 1x add layer with 2048 chanel
* flatten layer, dense layer 1 and dense layer 2

### Employed Statistical Performance Metrics

In DL based classification or disease diagnosis studies, the performance of the model must be evaluated with performance measurement metrics. Otherwise, the classification or diagnostic work performed will be deprived of quantitative evaluation. The statistical metrics, which have been employed in the proposed system have been derived from confusion matrix and can be calculated using the following equations. For example, accuracy is the percentage of samples classified as correct instances. Recall is a metric that shows how many of the instances that should be positively predicted are positively predicted whereas precision shows how many of the positively predicted values are actually positive. F1-score is a measure of the precision and robustness of the model.

(2) $$\mathrm{Accuracy}\;(\text{\%})=\frac{TP+TN\;\;}{TP+TN+FP+FN}\times 100$$

(3) $$\mathrm{Precision}=\frac{TP}{TP+FP}$$

(4) $$\mathrm{Recall}=\frac{TP}{TP+FN}$$

(5) $$F1-\mathrm{score}=\frac{2TP}{2TP+FP+FN}$$

where TP, TN FP, and FN stand for true positives, true negatives, false positives, and false negatives, respectively.

### Monte-Carlo Cross-Validation

Monte Carlo cross-validation^34^ is a method used in data modelling to evaluate the performance of a model. It differs from classical cross-validation in that it repeatedly tests the model by generating different random partitions each time. It works as follows:

1. Partition the data: The data set is randomly partitioned into training and test sets in a certain proportion (eg 70% training, 30% test).
2. Train the model: The model is trained on the training set.
3. Evaluate the model: The performance of the model is evaluated on the test set.
4. Repetition: This process is repeated a certain number of times (eg 1000), each time randomly dividing the data set into different parts.

This method can provide more reliable results for understanding the overall performance of the model because it is evaluated more than once on different partitions. In this study, the Monte Carlo cross-validation method was employed.

## Results

The experiments have been performed on Intel (R) Core (TM) i9-1085K CPU @ 3.60 GHz and GeForce RTX3080 graphical process unit hardware environment. The software environment for the experiments were Windows 10 (64-bit) operating system and Keras library in Python. The experiments have been conducted each time using MobileNet, VGG16 and ResNet50 CNN models, respectively. In all experiments, the prepared data have been divided into training set (%64), validation set (%16) and test set (%20). To improve the performance and generalization capabilities of the models and to decrease the potential sources of bias such as the representativeness of the dataset, Shuffle Algorithm with seed value 1 has been used for dataset division (training, validation, and test). Table 5 shows the details of learning scheme of each CNN model. Each CNN model has been trained on a total of 16 000 histopathological images and validated on 4000 histopathological images. Finally, the trained model has been tested using 5000 test images. Hence, for each image group, 3200 images have been used for training, 800 images have been used for validation and 5000 images have been used for testing the model. It is worth noting that 20% ratio has been selected for the test. However, due to the shuffle seed (randomly generated number), the number of tests taken for each class is different. The performance of each model has been evaluated using accuracy, precision, recall and F1-score. Taking the practical application of this study into account, the computational requirements are given above. Besides the runtime of the proposed system are as follows. 20 epochs have been performed to train the model and each epoch has taken 165 s and 326 milliseconds. A 55 min and 10 s duration has been required to train the model. The testing stage has taken 7 min and 6 s. Therefore, the total runtime of the proposed system is 1 h 2 min and 16 s.

**Table 5.** Learning Scheme of Each CNN Model.

Classification Groups	Training Set (64%)	Validation Set (16%)	Test Set (20%)
Colon Adenocarcinoma	3200	800	1000
Benign Colonic Tissue	3200	800	1000
Lung Adenocarcinoma	3200	800	1000
Benign Lung Tissue	3200	800	1000
Lung Squamous Cell Carcinoma	3200	800	1000
**Total**	16 000	4000	5000

**Figure 4.**
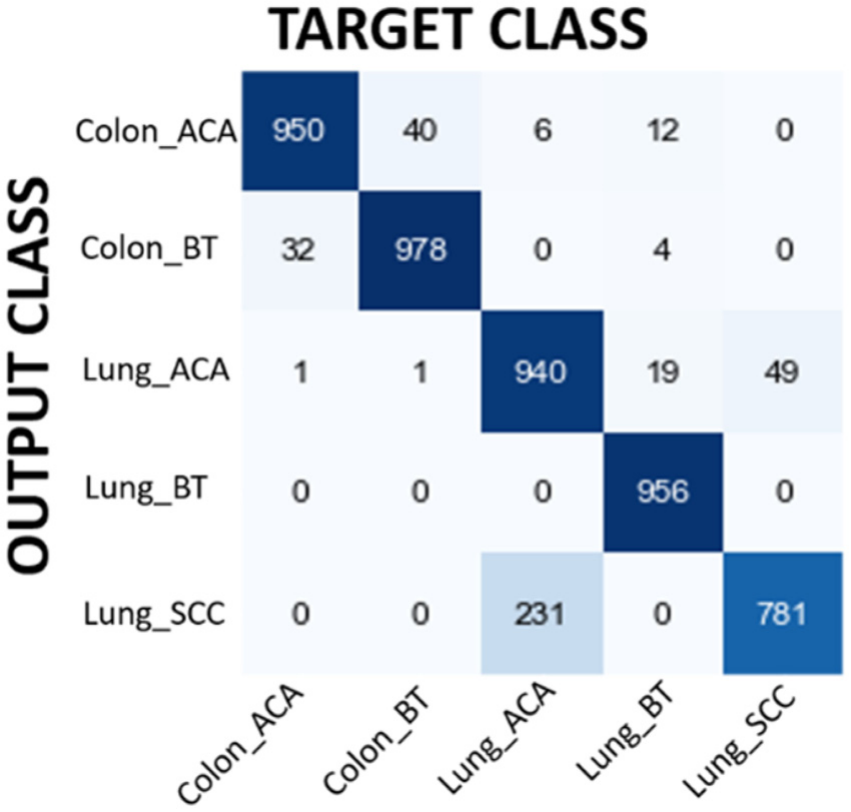
Confusion matrix results for MobileNet model.

In the first experiment MobileNet has been used to classify input image as Colon Adenocarcinoma, Colon Benign Tissue, Lung Adenocarcinoma, Lung Benign Tissue and Lung Squamous Cell Carcinoma. Table 6 shows the performance evaluation of MobileNet. The overall accuracy has been found as 92.10% which is quite promising for lung and colon cancer detection. The results demonstrated in Table 3 show the power of MobileNet in detecting lung and colon cancers from histopathological images using the idea of DL approach. The performance of the MobileNet model has been also evaluated using confusion matrix. Figure 4 shows the confusion matrix obtained for MobileNet model. When confusion matrix is analyzed in detail, it is understood that the model's classification ability is pretty good except Lung Adenocarcinoma and Lung Squamous Cell Carcinoma classification. The model classifies 231 Lung Adenocarcinoma histopathological images as Lung Squamous Cell Carcinoma. On the other hand, the model classifies 49 Lung Squamous Cell Carcinoma histopathological images as Lung Adenocarcinoma. In addition to this, 40 Colon Benign Tissue histopathological images are misclassified as Colon Adenocarcinoma and 32 Colon Adenocarcinoma histopathological images are misclassified as Colon Benign Tissue. The model seems to have a high success rate in the remaining groups. These results also show that the model can confuse histopathological images belonging to the same organ, which is an expected result. Although filtering contributed to the improvement of the results, it would be interesting to evaluate how many images lose their original information (class change) after filtering. For instance, 22 Colon Benign Tissue images lose their original class after filtering. Moreover, 123 Lung Squamous Cell Carcinoma lose their original class.

**Table 6.** Model Statıstıcal Performance Evaluation Results for MobileNet Model.

Classification Groups	Precision	Recall	F1-score	Support
Colon Adenocarcinoma	0.94	0.97	0.95	983
Colon Benign Tissue	0.96	0.96	0.96	1019
Lung Adenocarcinoma	0.93	0.80	0.86	1177
Lung Benign Tissue	1.00	0.96	0.98	991
Lung Squamous Cell Carcinoma	0.77	0.94	0.85	830
Accuracy (%)	—	—	**92**.**10**	
**Average**	0.92	0.93	0.92	

**Figure 5.**
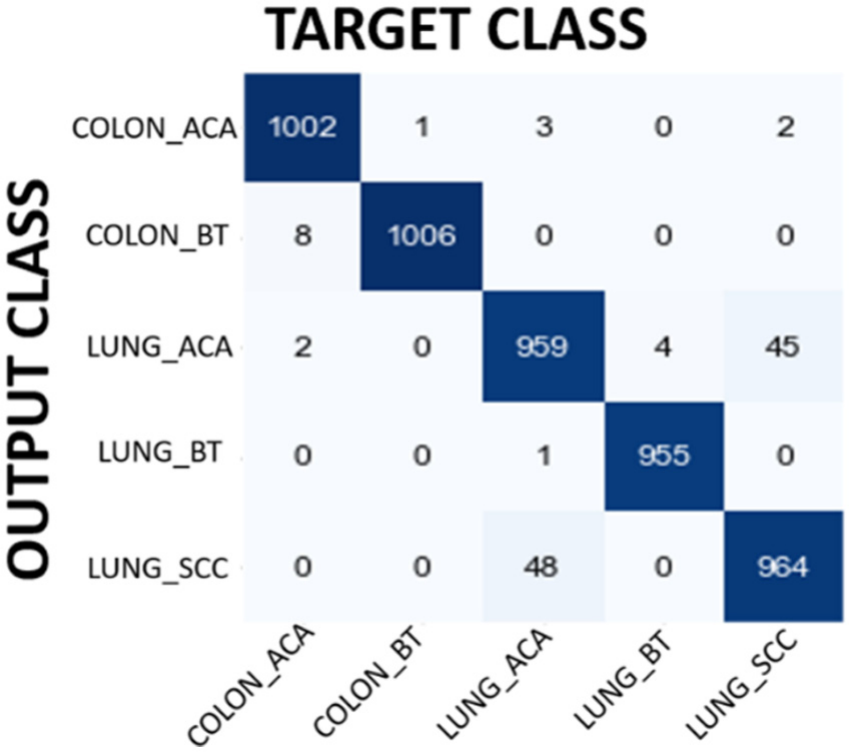
Confusion matrix results for VGG16 model.

In the second experiment, the same classification study has been performed using VGG16 model. Table 7 shows the performance evaluation of VGG16. The overall accuracy has been found as 97.72 which is better than the results of MobileNet. Figure 5 shows the confusion matrix obtained for VGG16 model. The model classifies 48 Lung Adenocarcinoma histopathological images as Lung Squamous Cell Carcinoma. On the other hand, the model classifies 45 Lung Squamous Cell Carcinoma histopathological images as Lung Adenocarcinoma. These results clearly indicate that the model has trouble distinguishing between Lung Adenocarcinoma and Lung Squamous Cell Carcinoma groups. The model seems to have a high success rate in the remaining groups. It is noteworthy here that the VGG16 model is able to distinguish between Colon Adenocarcinoma and Colon Benign Tissue more successfully, unlike the MobileNet model. However, the results also show that the model can still confuse histopathological images belonging to the same organ (Lung Squamous Cell Carcinoma vs Lung Adenocarcinoma). When we evaluate how many images lose their original class, as we did in MobileNet model, we see that 3 Colon Benign Tissue images lose their original class after filtering. Moreover, 13 Lung Squamous Cell Carcinoma lose their original class.

**Table 7.** Model Statıstıcal Performance Evaluation Results for VGG16 Model.

Classification Groups	Precision	Recall	F1-score	Support
Colon Adenocarcinoma	0.99	0.99	0.99	1012
Colon Benign Tissue	0.99	1.00	1.00	1007
Lung Adenocarcinoma	0.95	0.95	0.95	1011
Lung Benign Tissue	1.00	1.00	1.00	959
Lung Squamous Cell Carcinoma	0.95	0.95	0.95	1011
Accuracy (%)	—	—	**97**.**72**	
**Average**	0.98	0.98	0.98	

**Figure 6.**
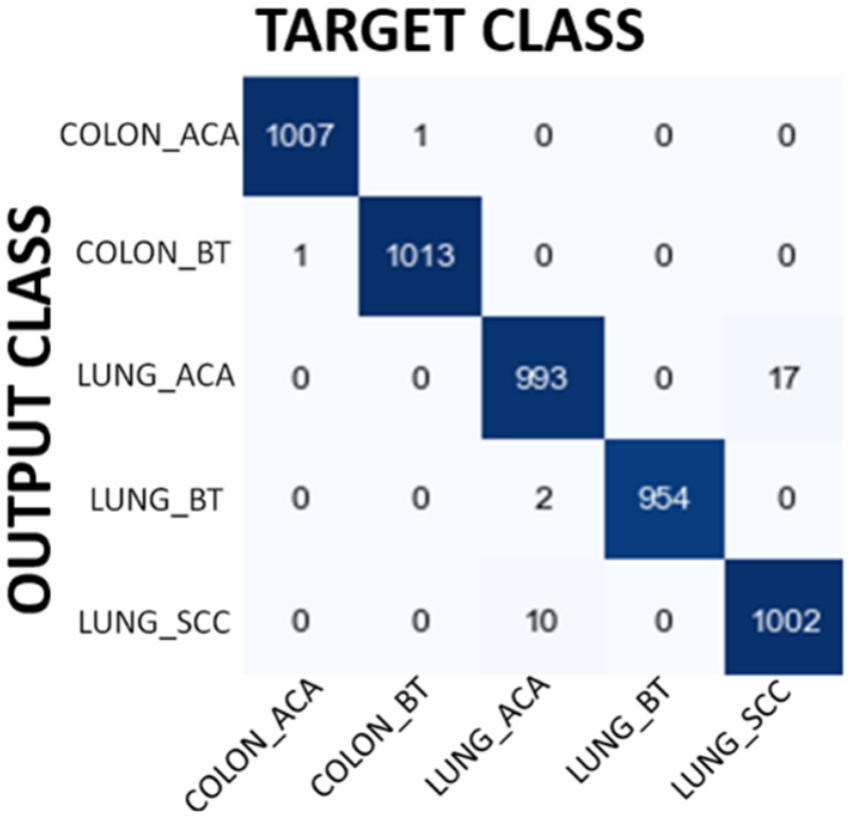
Confusion matrix results for ResNet50 model.

In the third experiment, ResNet50 CNN model has been used to classify the images. Overall accuracy obtained with this model is 99.38% which is even better than the results obtained by VGG16 and MobileNet model. The details of the performance results obtained with ResNet50 are shown in Table 8. Figure 6 shows confusion matrix results for ResNet50 model. Confusion matrix shows that the ResNet50 model achieves high classification success in groups where the success level of MobileNet and VGG16 models is relatively low. Therefore, ResNet50 model shows high classification success in all groups, including groups belonging to the same organ. It is again interesting that 1 Colon Benign Tissue image loses its original class after filtering. Similarly, 1 Lung Benign Tissue image loses its original class after filtering.

**Table 8.** Model Statıstıcal Performance Evaluation Results for RESNET50 Model.

	Precision	Recall	F1-score	Support
Colon Adenocarcinoma	1.00	0.99	1.00	1012
Colon Benign Tissue	0.99	1.00	1.00	1010
Lung Adenocarcinoma	0.98	0.98	0.98	1009
Lung Benign Tissue	1.00	1.00	1.00	958
Lung Squamous Cell Carcinoma	0.98	0.98	0.98	1011
Accuracy (%)	—	—	99.38	
**Average**	0.99	0.99	0.99	

**Figure 7.**
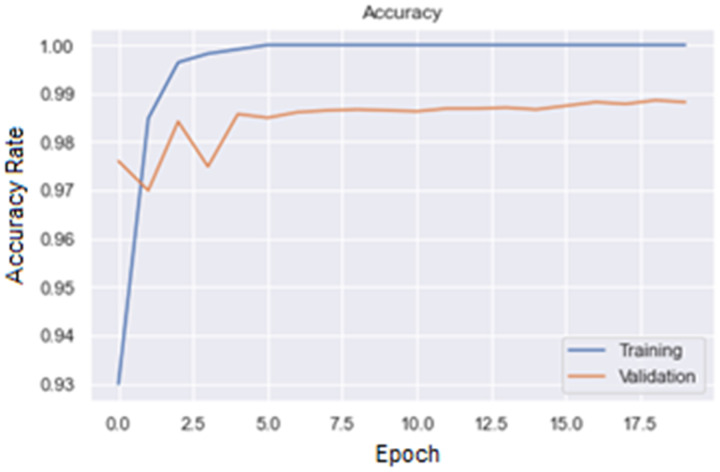
Accuracy plot for ResNet50 model.

**Figure 8.**
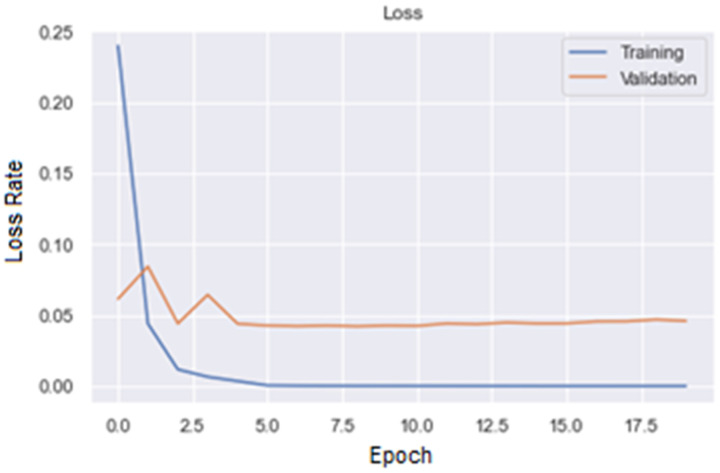
Loss plot for ResNet50 model.

Figures 7 and 8 are, respectively, plot of Accuracy and Loss obtained with the ResNet50 model to classify images.

**Figure 9.**
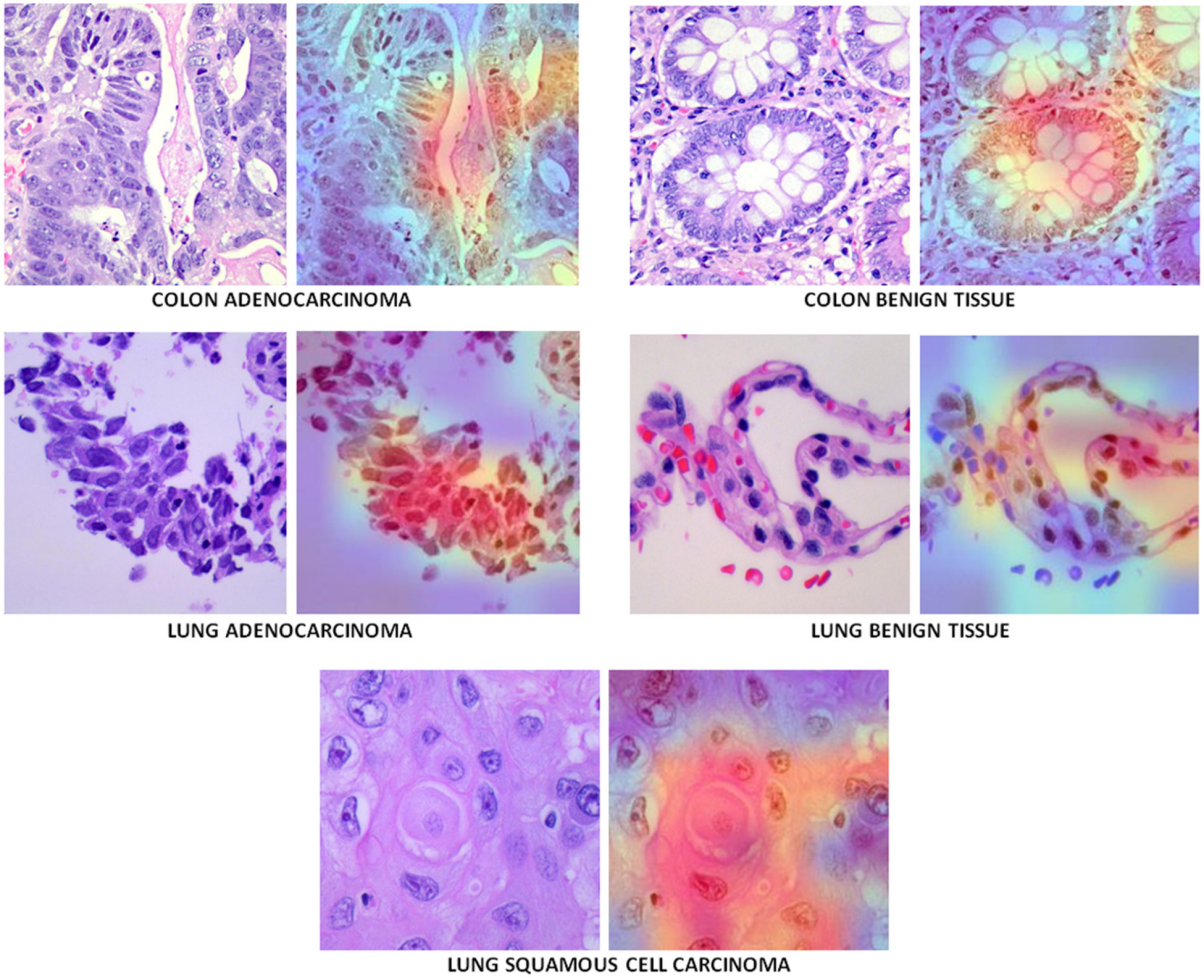
Class activation map (CAM) visualization results.

In order to ensure that the classification made is reliable and valid, it becomes very easy to determine which regions of the image-based input data are used in the decision phase through the heat maps obtained thanks to the use of the Class Activation Map (CAM) tool. CAM tool has been used to see the parts of the image that the CNN model was particularly interested in and learned from. The CAM tool gives the researcher visual information about where and how the network has learned, visually revealing the parts the network has actually learned. Figure 9 is CAM visualization results for each classification group. As figure suggests, CAM allows the network to show the visual regions it focuses on to the user by displaying it in heat map format. Hence, as the performance evaluation metrics prove with numerical data that the DL network learns, CAM visually proves that the DL network learns. In this study, CAM has been integrated into the CNN model as it is capable of providing information on whether the input images are particularly focused on cancerous regions.

In the proposed study, 2D Gaussian Blur filter has been suggested as a pre-processing step to increase the learning ability of DL models by removing undesired noises such as: dots and disturbances from images that may cause false learning of DL models. All the experiments have been performed with and without filter to demonstrate the effect of filtering. Table 9 shows the overall accuracy results for each model (MobileNet, VGG16 and ResNet50) with and without 2D Gaussian filter. As can be seen from the table, filtering increases the result from 88.63% to 92.44% for MobileNet, from 92.57% to 98.23% for VGG16 and finally from 96.54% to 99.38% for ResNet50.

**Table 9.** Accuracy Results of Each Model with and Without Filter.

	Accuracy Results (%)	
	Without Filter	With 2D Gaussian Filter
MobileNet	88.63	92.10
VGG16	92.57	97.72
ResNet50	96.54	99.38

The state-of-the-arts results from the recent studies announced in the literature are summarized in Table 10. To begin with, Shi et al used a novel method so called multimodal sparse representation-based classification technique to diagnose lung cancer using 271 number of needle biopsy images and achieved 88.1% classification accuracy.^9^ The authors did not perform any experiments on colon cancers types. Besides, the number of images in dataset is not high enough for a reliable diagnostic system. Kuruvilla et al proposed neural network method for lung cancer diagnosis from CT images of 155 patients and obtained an overall accuracy of 93.3%.^10^ Similarly, this study only investigated lung cancer detection and no experiment were performed for colon cancer diagnosis. In addition, the number of images used to train the neural network is not efficient for a learning algorithm. Selvanambi suggested using recurrent neural network (RNN) based on glowworm swarm optimization to diagnose lung cancer from benchmark dataset and no information was given on colon cancer diagnosis.^12^ Naeem et al made use of genomic signal processing to colon cancer diagnosis from DNA sequences and got an accuracy rate of 95%.^13^ In this study, lung cancer diagnosis was not investigated. Masud et al used CNN network approach to classify lung and colon cancer histopathological images using 25 000 images and achieved an overall accuracy of 96.33%.^14^ One of the shortcomings of this study is that the classification success is low for two disease types (Lung Adenocarcinoma and Lung Squamous Cell Carcinoma) compared to the others. Another limitation is that two separate algorithms such as Discrete Fourier transform (DFT) and Discrete Wavelet transform (DWT) were used to extract features from histopathological images. These algorithms require high computational requirements and long runtime in practice. Togacar et al used DarkNet-19 CNN model and obtained a classification accuracy of 99.69% to diagnose lung and colon cancer using 25 000 histopathological images.^15^ Although the optimization algorithm was used to increase the classification success in this study, the success rate obtained is almost the same as the success rate of our proposed study, but there is no doubt that the optimization algorithm used will increase the hardware requirements in practical terms. Ali et al suggested Multi-Input Dual-Stream Capsule Network for Improved Lung and Colon Cancer Classification using 25 000 histopathological images and achieved 99.58% classification accuracy.^16^ The fact that two convolutional layer blocks were used and that the proposed model was given two inputs at the same time (one with original images and the second with pre-processed images) have made the overall system more complicated, which remains as a limitation of that study. Mehmood et al proposed malignancy detection in lung and colon histopathological images (25 000) using transfer learning with class selective image processing and obtained 98.4% accuracy.^19^ The well-known limitations of transfer learning approach such as limited flexibility, overfitting and limited generalization were not discussed in this study and still remains as the limitations of this study. Yu et al performed colorectal cancer diagnosis using semi-supervised DL on 13 111 pathological images and obtained an accuracy rate of 96.9%.^35^ However, in this study the classification of colon cancer types (Colon Adenocarcinoma and Colon Benign Tissue) were not included. In another study of classification of lung and colon cancers, Mridha et al achieved a classification accuracy of 98.3% using four different CNN models, each having a separate classification problem.^20^ The problem with this study is that a binary classification was performed for a multi-class problem. In addition, another shortcoming of the proposed method is the high tendency of colon scans to give false positives. Teramoto et al have designed a novel CNN model for lung cancer diagnosis and obtained a classification accuracy of 71%. The fact that the success rate in this study is not high and that the diagnosis of colon cancer has not been made are the important shortcomings of this study.^21^ Kumar et al performed lung and colon cancer diagnosis using two feature extraction strategies. They obtained 98.6% classification accuracy by using DenseNet-121 CNN model for feature extraction and Random Forest technique for the classification. One important shortcoming of this study is that the computational power requirement and training time have been increased because RF classifier has to produce and combine many decision trees.^22^ As the literature shows, when the proposed study is compared with other studies in the literature it is possible to say that the proposed method is highly promising in accurately diagnosing lung and colon cancer with a high rate of success.

**Table 10.** The Comparison of the Proposed Method with the Recent Studies in the Literature.

Reference Study	Method Used	Diagnosis Type	Dataset Used	Image Number	Accuracy (%)
^9^	mSRC	Lung Cancer	Bayi Hospital	271	88.1
^10^	Neural Network	Lung Cancer	Lung Image Database Consortium	155	93.3
^12^	RNN	Lung Cancer	UCI repositories	32	98
^13^	KNN-SVM	Colon Cancer	NCBI GenBank	110	95
^35^	SSL	Colon Cancer	Multi-center	13 111	96.9
^36^	RF	Colon Cancer	Aster Medcity	-	85.3
^37^	CNN	Lung and Colon Cancer	Lung Image Database Consortium	1279	97.9
^14^	CNN	Lung and Colon Cancer	LC25000	25 000	96.33
^15^	CNN	Lung and Colon Cancer	LC25000	25 000	99.69
^38^	Hybrid CNN	Lung and Colon Cancer	LC25000	25 000	99.30
^16^	CNN	Lung and Colon Cancer	LC25000	25 000	99.58
^19^	CNN with CSIP	Lung and Colon Cancer	LC25000	25 000	98.4
^20^	CNN	Lung and Colon Cancer	LC25000	25 000	98.3
^21^	CNN	Lung and Colon Cancer	HM16-155	298	71.3
^22^	CNN-RF	Lung and Colon Cancer	LC25000	25 000	98.6
**Proposed method**	**MobileNet, VGG16, ResNet50**	**Lung and Colon Cancer**	**LC25000**	**25 000**	**99**.**38**

Currently, the most effective and therefore, preferred diagnostic method for lung and cancer detection is medical imaging and the analysis of these images by experts such as doctors or radiologists. The complexity of medical images and especially the huge number of examinations by human experts leads to misdiagnoses. Therefore, accurate lung and colon cancer detection remains a challenging task. CAD systems continue to yield highly successful results in diagnosing medical diseases.

## Conclusion

Computer-Aided Diagnosis (CAD) systems have been proven to be very successful and promising in minimizing misdiagnoses. In this study, the CNN technique has been modeled to be used for the detection of lung and colon cancer, and the safety level of the model has been presented in an explanatory via the Class Activation (CAM). The proposed CAD system in this paper is able to diagnose lung and colon cancer with a high accurate rate such as 99.38%. The proposed method outperforms existing methods significantly, considering the results of the proposed method and the results of comparison with similar studies in the literature. The CAD approach proposed in this study is believed to have the potential of assisting the doctors and physicians to verify their lung and colon cancer diagnosis decisions.
